# Depression and Chronic Liver Diseases: Are There Shared Underlying Mechanisms?

**DOI:** 10.3389/fnmol.2017.00134

**Published:** 2017-05-08

**Authors:** Xiaoqin Huang, Xiaoyun Liu, Yongqiang Yu

**Affiliations:** ^1^Department of Psychiatry, The First Affiliated Hospital of Anhui Medical UniversityHefei, China; ^2^Department of Radiology, The First Affiliated Hospital of Anhui Medical UniversityHefei, China

**Keywords:** chronic liver disease (CLD), depression, hepatitis, non-alcoholic fatty liver disease (NAFLD), alcoholic liver disease (ALD)

## Abstract

The occurrence of depression is higher in patients with chronic liver disease (CLD) than that in the general population. The mechanism described in previous studies mainly focused on inflammation and stress, which not only exists in CLD, but also emerges in common chronic diseases, leaving the specific mechanism unknown. This review was to summarize the prevalence and risk factors of depression in CLD including chronic hepatitis B, chronic hepatitis, alcoholic liver disease, and non-alcoholic fatty liver disease, and to point out the possible underlying mechanism of this potential link. Clarifying the origins of this common comorbidity (depression and CLD) may provide more information to understand both diseases.

## Introduction

Chronic liver disease is the progressive destruction and regeneration of the liver parenchyma leading to fibrosis and cirrhosis (normally lasts 6 months). The etiologic agents of CLD include hepatotropic viruses (HBV and HCV), fatty liver, alcohol, autoimmune hepatitis, etc. The mortality of CLD patients remains high not only due to irreversible cirrhosis, but also multiple complications such as HCC and mental disease, especially depression.

Depression, also named MDD, MDE, or clinical depression, is a mental disorder characterized by a pervasive and persistent low mood that is accompanied by low self-esteem and by a loss of interest or pleasure in normally enjoyable activities. CLD has been long recognized and associated with depression (Gutteling et al., 2006; Patten et al., 2008). Clinical studies have shown that some patients with CLD have more severe depressive tendencies (Mullish et al., 2014) than healthy ones. However, the underlying mechanisms of this association remain largely unknown, since previous studies used different diagnostic criteria to evaluate depression in chronic diseases. For example, Ismail et al. (2007) used standard psychiatric interview (Schedules of Clinical Assessment in Neuropsychiatry) to diagnose MDD in patients with diabetes and their first foot ulcer. In a cross-sectional study, Popović et al. (2015) used the Hamilton depression rating scale (HDRS) in patients with CLD and found that depression was present in 62.9% of them. In addition, a number of studies on CLD have instead used self-report questionnaires (Bellentani et al., 1994; Dwight et al., 2000; Dan et al., 2006).

Here, we summarize studies that used both conventional diagnostic criteria and questionnaires to identify depression in CLD [especially in viral hepatitis, ALD, and NAFLD], to point out the potential link between two diseases, which may provide more information to understand both diseases.

## The Link Between Depression and Cld

### Chronic Hepatitis B

Chronic HBV infection is defined as the persistence of HBsAg and serum HBV–DNA levels detectable for at least 6 months. Globally, CH-B affects approximately 400 million people worldwide and contributes to more than one million deaths annually of liver disease (Lavanchy, 2004). CH-B has become one of the major causes of chronic hepatitis, cirrhosis, and HCC worldwide. For patients with active viral replication, cirrhosis will be observed in 15–20% of patients within 5 years. Among patients with cirrhosis, the disease may progress, and the incidence of HCC is significantly elevated. 70–90% of patients with HCC have cirrhosis (Karayiannis and Thomas, 2005). It has been found that hepatitis B has a markedly negative impact on HRQOL, both in terms of physical function and mental health.

In 1984, Lok and his colleagues firstly studied the psychosocial effect of chronic HBV infection in 40 British carriers by using their own questionnaire. Thirty-six of these patients (90%) considered themselves that their physical and psychological health including sexual, work, social, and family attitudes, were negatively affected. Thirty-four of them experienced depression, 22 felt isolated especially initially, and 15 had feelings of guilt. In addition, 3 had sought, and 4 considered seeking psychiatric help (Lok et al., 1985). Although the sample size was small, this study indicates a potential relationship between CLD and depression. Consisting with this finding, a couple of years later, Kunkel et al. (2000) evaluated 50 Korean immigrants who had CH-B or were healthy carriers for the HBV in terms of the relationships between their depression scores, psychosocial stressors, social support, and biological markers of dysfunction. All patients completed the BDI-sf (Hojat et al., 1986), a self-report BDI screening test for depression that is scored as an index of severity of depressed mood. The average total BDI-sf score of them was 5.4 (BDI-sf scores >5 means mild to severe depression). Twenty-three patients (46%) endorsed depressive symptomatology. Interestingly, higher depression scores were significantly associated with transaminase elevations both before and after BDI-sf administration, which discloses a more concrete relationship between depression and CLD than Lok’s study, regardless their similar sample size and absence of controls. This limitation was much improved by a larger study from Weinstein et al. (2011) in which the prevalence of depression was compared among patients with CH-B, CH-C, and NAFLD based on patient self-report and confirmed by examining history of prescription medication. In this study, a total number of 190 CH-B were enrolled, and only 7 (3.7%) had a diagnosis of depression (Weinstein et al., 2011). The low morbidity of depression in this study may be attributed to the missing self-report by the patients who had mild or moderate depression.

Actually, not only CH-B patients, but also HBsAg carriers need psychological therapies (**Table 1**). Atesci et al. (2005) studied the psychiatric morbidity in a homogeneous group of asymptomatic HBV carriers by using the BDI, DSM IV Axis I (psychiatric disorders) and Axis V (global functioning assessment). They found that HBV carriers were more likely to have psychiatric disorders than healthy subjects (30.2% vs. 11.6%). Furthermore, carriers had significantly higher depression scores and lower GAF scores than comparison subjects. The high rates of psychiatric disorder in HBV carriers might be related to the frightening and potentially stigmatizing nature of hepatitis B (Atesci et al., 2005). However, a Turkish study examining the effect of HBV infection on the psychological state of children showed no significant difference between asymptomatic carrier patients and control children with respect to depression scores. The different result may be explained by: (i) the absence of symptoms and insufficient information about children’s status; (ii) the society protecting children from stigmatization (Arslan et al., 2003). These possible explanations suggest that children are a special group and are not appropriate to compare with adults. Nevertheless, another study showed that the prevalence of depression increased significantly among CH-B patients in Iran (29.3%) after excluding inactive HBsAg carriers (10.0%), whereas it was similar between inactive HBsAg carriers and healthy participants (10.0% vs. 11.3%) (Mirabdolhagh Hazaveh et al., 2015). In contrast, Altindag et al. (2009) reported that 20% of CH-B patients were suffering from severe depression (score ≥ 17), and this number fell to 13.3 and 3.3% in HBsAg carriers and healthy controls, respectively. In addition, HRQOL scores in asymptomatic carriers were similar to those of non-cirrhotic CH-B patients, but worse than those of healthy controls (Altindag et al., 2009).

**Table 1:** The prevalence of depression in patients with CH-B.

Patients	Methods	Results	Reference
40 British HBV carriers	Questionnaire	34 patients experienced depression 22 felt isolated 15 felt guilty 3 had sought and 4 considered seeking psychiatric help	Lok et al., 1985
50 Korean CH-B Healthy HBV carriers	Clinical interview BDI-sf	23 patients (46%) endorsed depressive symptomatology	Kunkel et al., 2000
20 CH-B children patients 20 asymptomatic carriers 43 healthy control	CDI	No difference between asymptomatic carrier and control group children with respect to depression scores	Arslan et al., 2003
43 asymptomatic carriers 43 healthy controls	BDI-sf Structured Clinical Interview for DSM-IV GAF Scale	HBV carriers were more likely to have psychiatric disorders than comparison subjects (30.2% vs. 11.6%) Carriers had significantly higher depression scores and lower GAF scores than comparison subjects	Atesci et al., 2005
30 CH-B patients 30 inactive HBsAg carriers 30 healthy controls	BDI-sf	20% of CH-B patients were suffering from severe depression, 13.3% in HBsAg carriers, and 3.3% in healthy controls	Altindag et al., 2009
190 CH-B patients	Self-report Examine history of prescription medication	3.7% CH-B patients had a diagnosis of depression	Weinstein et al., 2011
81 Iran CH-B patients 80 healthy controls	HAMD-17	Inactive HBsAg carriers and CH-B patients-excluding inactive HBsAg carriers-differed significantly in their prevalence of depression (10.0% vs. 29.3%)	Mirabdolhagh Hazaveh et al., 2015

*BDI-sf, Beck Depression Inventory Short Form; CDI, Children’s Depression Inventory; DSM, Diagnostic and Statistical Manual of Mental Disorders; GAF, Global Assessment of Functioning; 17-item Hamilton Depression Rating Scale (HAMD-17); HRQOL, health-related quality of life.*

### Chronic Hepatitis C

Chronic hepatitis C is a worldwide health problem that affects approximately 170 million people globally (3%) (Lavanchy, 2011; Mohd Hanafiah et al., 2013). CH-C is now the most common cause of end-stage liver failure and the leading indication for liver transplant in the developed countries. Emerging evidence suggests that psychiatric problems, particularly depression, frequently occur in chronic infection with HCV and during antiviral treatment. As in other chronic medical illnesses, the presence of depression symptoms in CH-C is critical because they exhibit an adverse effect on the course of illness, with amplification of physical symptoms, functional impairment, and decreased compliance of treatment and quality of life (Dwight et al., 2000).

High proportion of patients with CH-C had been diagnosed as having depression (Schaefer et al., 2012) (**Table 2**). For example, Dwight et al. (2000) evaluated 50 CH-C patients using structured psychiatric interviews and standardized rating instruments, and found that 14 (28%) of patients had current depressive disorders, and their disability and fatigue were more closely related to depression severity than to hepatic disease severity. In another large study of 22341 HCV-infected veterans and 43267 healthy controls, el-Serag et al. (2002) reported that patients were more likely to have depressive disorders than controls (49.5% vs. 39.1%). A recent study assessing the association between different types of CLD with depression in a population-based cohort showed that depression was strongly associated with CH-C (Lee et al., 2013). Furthermore, Golden et al. (2005) studied 90 CH-C participants and demonstrated a 28% 1-month prevalence of depression, and 72% of whom were previously unidentified. Carta et al. (2007) provided the evidence that CH-C was associated with MDD, independent of IFN-α treatment (32.6%).

**Table 2:** The prevalence of depression in patients with CH-C.

Patients	Methods	Results	Reference
50 CH-C patients	Structured psychiatric interview DSM IV BDI-sf21	14 (28%) of patients had current depressive disorders	Dwight et al., 2000
22341 CH-C veteran patients 43267 controls	ICD-9 CM	CH-C patients were more likely to have depressive disorders than healthy controls (49.5% vs. 39.1%)	el-Serag et al., 2002
90 CH-C patients	Structured Clinical Interview for DSM-IV Axis I Disorders	There was a 28% 1-month prevalence of depressive disorders, 72% of whom were previously undiagnosed.	Golden et al., 2005
135 CH-C patients, 540 healthy control	DSM-IV	A significantly higher number of lifetime diagnoses of MDD among patients with CHC compared to controls (32.6% vs 12.8%), independent of IFN-α treatment.	Carta et al., 2007
162 CH-C at baseline and treatment with IFN	20-item SDS	Compared with baseline, mean SDS index scores were significantly increased by week 4 and remained elevated throughout the study. 39% of patients experienced moderate to severe depressive symptoms at some point during the therapy.	Schaefer et al., 2002
325 CH-C patients	MDI/DSM-IV criteria	19 patients (6%) had major depression at baseline and an additional 114 (37%) developed depression while on HCV combination therapy. Only 36 (32%) of the 114 patients developed major depression.	Leutscher et al., 2010

*DSM, Diagnostic and Statistical Manual of Mental Disorders; BDI-sf, Beck Depression Inventory Short Form; ICD-9-CM, Clinical Modification of the International Classification of Diseases; SDS, Zung Self-Rating Depression Scale; MDI, Major Depression Inventory.*

### Alcoholic Liver Disease

Chronic excessive alcohol use is a common global health care concern and is associated with significant high morbidity and mortality. Approximately 20 to 30% of primary LTs are performed for patients with ALD (Erim et al., 2007; Adam et al., 2012; Stepanova et al., 2014). Alcohol use disorders are the second common indication for LTs in the US and Europe (Burra et al., 2010; Rice et al., 2013; Singal et al., 2013). The term “ALD” comprises a spectrum of pathologic characteristics including isolated steatosis, alcoholic steatohepatitis and established cirrhosis. Within this spectrum, varying degrees of inflammation, ballooning degeneration and hepatocyte necrosis, cholestasis, and fibrosis may be encountered (Sakhuja, 2014).

Some researchers proposed that alcohol use may lead to depression symptoms as well as an increased risk of parasuicide and suicide (Gilman and Abraham, 2001; Cargiulo, 2007). In contrast, others have found that depression may escalate alcohol use (Gilman and Abraham, 2001; Crum et al., 2005). Interestingly, a recent study showed that depression increased the risk of alcohol dependence only in men (Bulloch et al., 2012). These findings suggest that ALD may be associated with mental morbidity as well. Unsurprisingly, further studies reported that ALD was associated with depressive symptoms, particularly in the elderly (Cargiulo, 2007; Seitz and Stickel, 2007; O’Shea et al., 2010). Given the aging of the US population, it is increasingly significant to evaluate the relationship between ALD and depression. In a cohort of patients with ALD including both cirrhotic and noncirrhotic, Ewusi-Mensah et al. (1983) showed that 40% of patients had psychiatric disorders, although there was no direct correlation between the degree of ALD and the likelihood of depression. In LT recipients with ALD, the psychologic stress from end-stage liver disease (Singh et al., 1997; DiMartini et al., 2011), surgical evaluation, and candidacy for LT (Dew et al., 1998), and subsequent surgery are risk factors in the development of depression (Corruble et al., 2011; DiMartini et al., 2011). Regarding the association between ALD and depression, further research should investigate the bidirectional relationship between them.

### Non-alcoholic Fatty Liver Disease

Non-alcoholic fatty liver disease is the hepatic independent manifestation of the metabolic syndrome. The spectrum of NAFLD ranges from simple steatosis through NASH and fibrosis to ‘cryptogenic’ cirrhosis, HCC and other complications (liver failure and gastroesophageal varices) (Clark et al., 2002; Youssef and McCullough, 2002; Hui et al., 2003). NAFLD is strongly associated with obesity and insulin resistance-related metabolic features such as type 2 diabetes mellitus, hyperlipidemia, and systemic hypertension (Wanless and Lentz, 1990; Bellentani et al., 1994; Clark et al., 2002; Charlton et al., 2011). With an aging and increasingly obese and diabetic population (Wild et al., 2004), the prevalence of NAFLD is significantly rising, leaving a public health concern among industrialized nations. Current estimates suggest that the prevalence in the general population is around one-third (Browning et al., 2004; Chitturi et al., 2004), rising in high-risk populations to as high as 70% (Angulo, 2002; Targher et al., 2007). As a primary cause, NAFLD has become the third most common cause of LTs in the US (8.5%) (Charlton et al., 2011).

There is growing evidence of links between NAFLD and depression. Several population-based studies have identified high prevalence of depression in patients with NAFLD. For instance, a study using a large population of CLD indicated that the prevalence of depression was significantly higher in patients with NAFLD compared with that in patients with CH-B (27.2% vs. 3.7%). They also found independent predictors of depression in this cohort were the presence of hypertension, smoking, history of lung disease, being female, and non-African–American. However, its clinical features were not explicitly stated (Weinstein et al., 2011). Another small matched case-control study by Elwing et al. (2006) showed that the prevalence of lifetime MDD was higher in the NASH population than that in control subjects matched for age, gender, BMI, and waist-hip ratio. Importantly, they found that the diagnosis of MDD tended to be associated with steatosis grade, after adjusting for other clinical confounders. In accordance with this, Youssef et al. (2013) also provided evidence that psychiatric disorders were associated with histological severity of liver diseases. They assessed a cohort of 567 North American patients with biopsy-proven NAFLD and demonstrated that subclinical and clinical depression were noted in 53 and 14% of NAFLD patients, respectively, Interestingly, depression was associated with more severe hepatocyte ballooning and patients with subclinical depression had a higher likelihood of having more severe portal fibrosis after adjusting for confounders (Youssef et al., 2013). These results are consistent with another recent study by Tomeno et al. (2015), in which NAFLD patients comorbid with MDD were characterized by more severe histological steatosis and higher NAFLD activity score. Moreover, serum aminotransferase, GGT, ferritin, and hs-CRP were dramatically higher in the NAFLD patients with MDD than those without MDD.

## The Effect of Antiviral Medications on Liver and Depression

Depression is a condition not only associated with HCV infection, but also with pegylated IFN-α treatment in patients with CH-C. IFN-α-associated depression develops in a total of 30–70% of HCV-infected patients during treatment, in which mild to moderate depression accounts for 45–60%, moderate to severe depression, 15–40%, and major depression, 15–45% (Raison et al., 2005a,b; Schaefer et al., 2002, 2003, 2007, 2012). The prevalence of IFN-α-associated depression is related to dosage and duration of treatment, and a previous history of psychiatric disorders (Schaefer et al., 2007; Leutscher et al., 2010). The prevalence depends on different assessment methods such as diagnostic interview, self-reports, clinical evaluation and observer-rated scales, and the severity of depression and the time of evaluation (Schaefer et al., 2012). Nevertheless, the type of IFN (pegylated or standard IFN), and combination therapy (IFN and RBV) have no significant impact on the prevalence of IFN-α-associated depression. A recent study by Leutscher et al. (2010) showed that 10% of CH-C patients treated with pegylated IFN and RBV had to prematurely withdraw from treatment, because 65% of them (most did not undergo antidepressant therapy) had developed major depression, which might adversely affect the outcome from HCV treatment. A number of earlier studies reported that anti-depressive treatment might optimize the outcome of HCV antiviral therapy, particularly by reducing the risk of early treatment discontinuation (Kraus et al., 2001; Maddock et al., 2004; Raison et al., 2005a). The recognition and treatment of pre-existing mental illness before HCV therapy yields improved tolerability and treatment response (Schaefer et al., 2007). Therefore, predicting psychiatric side effects of antivirus drugs is crucial for clinical research.

## Risk Factors of Depression in Patients with Ch-C

Given that some patients are more likely to present depression during treatment with IFN-α, researchers have increasingly focused on identifying various social, clinical and biological factors that may lead to depression symptoms (**Table 3**). The possibly substantial cultural differences, which exist between the societies in which the various quoted studies were conducted, might affect the risk of depression. Furthermore, stigmatization and the fact that CH-C patients have to fight with a chronic infectious disorder for a long time increase the occurrence of depression (Golden et al., 2005; Schafer et al., 2005). A previous history of depression was also demonstrated to be a risk factor for the development of depression during antiviral therapy (Hauser et al., 2002; Gohier et al., 2003; Raison et al., 2005a), while that was not observed in Leutscher’s study (Leutscher et al., 2010). Other clinical risk factors such as fatigue and sleep disturbance strikingly heralded the emergence of depression (Gohier et al., 2003; Leutscher et al., 2010). In addition, Horikawa et al. (2003) found that age was another apparent risk factor, although divergent observations in this regard have been reported previously by Hauser et al. (2002). They showed that there was no difference in age of CH-C patients between those who became IFN-induced MDD and those who did not. Similarly, some studies have yielded conflicting data with respect to female as a risk factor for the development of IFN-associated depression. Using multivariate regression analysis, Leutscher et al. (2010) identified female as a significant independent predictor for subsequent development of major depression. This finding is similar to previous study showing the greater likelihood of female patients to develop depression during IFN-α-2B and RBV treatment of CH-C (Fontana et al., 2002). However, Bonaccorso et al. (2002) found no significant gender-related differences in the effects of IFN-α on the development of depression.

**Table 3:** Potential risk factors of depression in patients with CH-C.

Social factors	Sociodemographic factors	Clinical factors	Genetic factors (Polymorphisms)
Cultural differences Stigmatization Long time stress	Age Female Ethnicity	A previous history of depression Fatigue Sleep disturbance	5-HTTLPR (the s allele of the short/long polymorphism) 5-HTR1A (C-1019G) IDO (rs9657182) IFNAR1 Immune genes (IL-6, TNF-α) Others (PLA2, COX2, epsilon4)

*5-HTTLPR, the serotonin transporter gene-linked polymorphic region; 5-HTR1A, the serotonin 1A receptor; IDO, indoleamine-2,3-dioxygenase; PLA2, Phospholipase A2; COX2, cyclooxygenase 2.*

## Evidence for Genomic Influence in Modulating Depression

Polymorphisms in specific genes encoding several key players of the serotonergic system have been shown to confer an increased risk for depression in HCV patients during IFN therapy (Capuron et al., 2003; Valentine and Meyers, 2005). Firstly, some studies have found the classical genetic risk factor for depression, the s allele of the short/long polymorphism within the 5-HTTLPR (Bull et al., 2009; Lotrich et al., 2009). Intriguingly, Pierucci-Lagha et al. (2010) investigated the functional role of these alleles (e.g., L_G_L_G_, L_G_S, and SS carriers were designated S’S’ and found low expression levels) and demonstrated that in Hispanic HCV patients with IFN-α treatment, S’ allele homozygotes showed markedly higher depression symptom scores. In contrast, among non-Hispanic Caucasians, L’ allele homozygotes showed more depression symptoms than S’ allele carriers did (Pierucci-Lagha et al., 2010). These findings suggest that ethnicity might be associated with this classical genetic risk factor during IFN therapy. Secondly, the 5-HTR1A is one of the most abundant receptors in the hippocampus and has been previously reported in some affective disorders (Naughton et al., 2000; Hensler, 2003; Navines et al., 2003; Lopez-Figueroa et al., 2004). Kraus et al. (2007) found that homozygosity for the G allele of the 5-HTR1A (C1019G) polymorphism in the promoter region of the 5-HT1A receptor gene conferred a higher risk for the development of IFN-induced depression compared to carriers with at least one C allele. In accordance with previous study, Cozzolongo et al. (2015) demonstrated that this C-1019G polymorphism within the transcriptional control region of the 5-HTR1A gene independently predicted the incidence of IFN-induced depression in patients with CH-C. Thirdly, relevant to the metabolism of serotonin, much more attention has been paid on IDO, which breaks down TRP, the primary amino acid precursor for serotonin. Although the IDO enzyme seems to be involved in the pathophysiology of IFN-induced depression, Galvao-de Almeida et al. (2011) suggested that there was no influence of the variants in the IDO gene and the diagnosis of IFN-related depression in the Brazilian population. Interestingly, Smith et al. (2012) found that in 800 Caucasians with CH-C, a polymorphism (rs9657182) in the promoter region of the gene encoding IDO was markedly associated with moderate or severe IFN-α-induced depression symptoms, whereas it did not predict depression in African Americans, who exhibited a significantly lower frequency of the risk allele at this locus (Smith et al., 2012). These observations confirm that ethnicity can modulate the risk for depression in CH-C during IFN therapy again. In addition to the serotonergic system, polymorphisms in the promoter region of the IFN-α/β receptor 1 (IFNAR1) can increase the risk of developing depression in HCV patients who are receiving antiviral treatments (Rifai and Sabouni, 2012). Besides, a growing number of studies have also reported an association between polymorphisms in immune genes and the incidence of depression (Gochee et al., 2004; Bull et al., 2009; Lotrich et al., 2010; Su et al., 2010).

## Shared Pathological Pathways

Although the reasons for the high prevalence of depression in patients with CH-C are not very clear, emerging evidence has supported the hypothesis that HCV directly or indirectly induces biological changes in the CNS, which may result in psychiatric symptoms. In a previous study using proton magnetic-resonance spectroscopy, Forton et al. (2001) demonstrated the elevations in basal ganglia and white matter choline/creatine ratios in patients with CH-C. Furthermore, the observation from Weissenborn et al. (2006) indicated the decreased alteration of serotonin and dopamine transporter binding in CH-C patients with chronic fatigue and cognitive impairment, which might be associated with depression. Of note, Grover et al. (2012) provided further *in vivo* evidence for a neurotropic role for HCV. Using PET with a ligand for microglial/brain macrophage activation, ^11^C-(R)-PK11195 (PK11195) and cerebral proton magnetic resonance spectroscopy, they demonstrated that microglial activation was positively correlated with HCV viraemia and altered cerebral metabolism in the brains of patients with CH-C (Grover et al., 2012). Therefore, HCV may have a specific biological role in the development of depression.

Activated innate immunity and an acute-phase inflammatory response are commonly implicated in pathogenesis of CLD. Hepatic inflammation promotes the development of liver fibrosis (LF), cirrhosis and HCC, in which inflammasomes may play a key role. Inflammasomes are multi-protein complexes, which can sense endogenous and exogenous danger signals, ultimately activating the inflammatory process. Accumulating evidence supports an important role of the NOD-like receptors (NLRs) family pyrin domain containing 3 (NLRP3) inflammasome in the initiation and progression of CLD (Henao-Mejia et al., 2012; Szabo and Petrasek, 2015). A recent study showed that HCV RNA engagement of the NLRP3 inflammasome stimulated IL-1β secretion in KCs (Negash et al., 2013). In addition to viral hepatitis, Wree et al. (2014b) demonstrated the NLRP3 inflammasome was an essential contributor to LF during NASH development in mice. Furthermore, NLRP3 inflammasome activation led to hepatocyte pyroptosis, severe hepatic inflammation and HSC activation accompanied by collagen deposition in the liver (Wree et al., 2014a). Interestingly, it has also been reported the relevance of the NLRP3 inflammasome in the pathogenesis of depression (Alcocer-Gómez et al., 2014; Choi and Ryter, 2014; Zhang et al., 2015). Alcocer-Gómez observed increased gene expression of NLRP3 and caspase-1 in blood cells, and increased serum levels of IL-1β and IL-18 in MDD patients (Alcocer-Gómez et al., 2014). Furthermore, the NLRP3 inflammasome was up-regulated in lipopolysaccharide-induced mice depressive-like behaviors (Zhang et al., 2014). Importantly, some drugs conferred an antidepressant effect in a model of depression by inhibiting NLRP3 inflammasome activation (Lu et al., 2014; Liu et al., 2015; Xue et al., 2015; Li et al., 2016). While the relationship of depression with CLD is complicated, shared biological mechanisms might underlie the association between depression and CLD in some individuals. The study focused on this underlying association will provide more information to understand both diseases. Overall, these findings indicate that the NLRP3 inflammasome is involved in the pathogenesis of depression and CLD, understanding its role in both diseases may build a linkage between them.

## Molecular Mechanisms and Targets Underlying a Possible Connection

All studies above mostly focused on the prevalence of depression in patients with CLD, while few studies investigated the underlying biological mechanism, especially the potential effect of HBV on brain and peripheral blood. Importantly, there are many limitations in early studies: (i) the sample size is small and there is no control; (ii) missing self-report by patients who have mild or moderate depression; (iii) the distribution of HBV infection worldwide varies according to geographical zone (Parkins et al., 2009). The impact of the carriage of HBV in highly epidemic (>8%) areas such as South East Asia and Africa is greatly different from low epidemic areas (<2%).

Recent advances have also been made to understand potential mechanisms leading to neuropsychiatric toxicity in CH-C patients treated with IFN-α. Numerous studies have indicated that significant alterations in serotonin, dopamine and norepinephrine neurotransmitter metabolism and activation of a pro-inflammatory cytokine network are involved in psychiatric disorders associated with IFN-α (Widner et al., 2000; Raison et al., 2005b; Neurauter et al., 2008; Capuron et al., 2011). Moreover, another mechanism underlying the psychiatric effects of IFN-α may involve alterations in neuroendocrine function. Raison et al. (2010). demonstrated that chronic exposure to IFN-α for 12 weeks in patients with CH-C was associated with a flattening of the diurnal ACTH and cortisol slopes, which might contribute to the altered diurnal HPA axis activity and behavior found in CH-C. However, in a previous study by Wichers et al. (2007), there was no relationship between daily average salivary cortisol concentrations or the cortisol awakening response and depression in HCV patients treated with IFN-α. In addition to neuroendocrine function, Fábregas et al. (2016) investigated whether there might be any specific alteration in plasma biomarkers associated with MD in patients with CH-C. They followed up 50 HCV-infected patients (14 of them treated with IFN), and found that the occurrence of MD increased significantly in patients during IFN treatment. Importantly, lower baseline adiponectin levels and use of IFN were associated with MD development, which indicates that adiponectin may be a resilience biomarker for MD in patients with CH-C (Fábregas et al., 2016). Accumulating evidence also supported an important role for decreased BDNF activity in the development of depression during IFN-α treatment (Lotrich et al., 2013). In summary, these evidences showed that the alterations in monoamine metabolism, impaired neuroendocrine function, and plasma biomarkers are the major aetiological pathways for IFN-induced neuropsychiatric symptoms in patients with CH-C, which may be helpful to improve the management of HCV patients receiving antiviral treatment and illuminate the IFN-induced depression.

Underlying mechanism between NAFLD and depression remains unknown. However, the production of pro-inflammatory cytokines, and the increased cortisol and epinephrine levels in patients with depression may be one plausible explanation for the link between depression and NAFLD (Maes et al., 1998; Miller et al., 2003; Penninx et al., 2003). Another potential explanation is the close correlation of NAFLD with obesity and diabetes mellitus, both of which have been strongly associated with depression symptoms (Lustman et al., 2000; Nouwen et al., 2010; Preiss et al., 2013; van Dooren et al., 2013; Muhlig et al., 2016). Nevertheless, this association cannot be fully explained by overweight status or poor adherence to therapy for diabetes alone, because anti-depressive treatment can improve insulin resistance and glycemic control (Lustman et al., 2000, 2005), which is independent of weight loss or adherence to diabetic treatment (Lustman et al., 1997, 1998; Okamura et al., 2000).

## Implications for the Clinical Management of Patients with Cld

Given the association of depression with severity of histological features of CLD, some investigators have focused on the influence of depression on the therapeutic effect of CLD. For example, Tomeno et al. (2015) compared the NAFLD clinical response within different stages of MDD, and suggested that NAFLD patients comorbid with MDD had poor response to the 48 weeks standard care for NAFLD. In particular, NAFLD patients with unstable MDD (not in full/partial remission) had severe resistance to the treatment (Tomeno et al., 2015). In addition to the therapeutic effect, Russ et al. (2015) performed a large sample meta-analysis to investigate the relationship between psychological distress (i.e., depression and anxiety) and liver disease (i.e., NAFLD and ALD) mortality using the 12-item GHQ. They found a significant increase in liver disease mortality with increase in GHQ score, which indicates that psychological distress is associated with mortality in CLD (Russ et al., 2015). Further research is needed to establish optimal management of depression in patients with NAFLD.

In addition to NAFLD, cirrhotic patients with depression experience worse health outcomes compared to matched patients without depression. In a previous study, Singh et al. (1997) assessed for orthotopic LT in depressed patients with decompensated liver disease of different aetiologies and found that these patients had an increased mortality at 100 days post-assessment compared to non-depressed patients. One possible explanation for this result is biological theories that focus on the effect of depression upon the immune system (dysregulated secretion of immunomodulatory cytokines and impaired lymphocyte function), ultimately may increase the propensity of patients with liver disease to decompensation (Koff et al., 1986; Irwin et al., 1987). Another explanation is psychological theories, which are based on the finding that depressed patients have high rates of noncompliance with medical regimens (DiMatteo et al., 2000).

Besides, as stated above, development of depression is the commonest reason for treatment discontinuation in patients with CH-C (Leutscher et al., 2010). Taken together, CLD or orthotopic LT patients with depression experience worse clinical outcomes, including a reduced quality of life and increased mortality, than those without.

## Conclusion

This review summarizes the information on the prevalence and various risk factors of depression in patients with CLD, and shared biological pathways, as well as potentially molecular mechanisms for their association. Patients afflicted with CLD including CH-B, CH-C, NAFLD, and ALD manifest different degrees of depression symptoms, which can adversely affect clinical care and patient outcomes. In addition, patients with cirrhosis had signs of psychological distress and depression in relation to the severity of liver disease (Bianchi et al., 2005). The majority of the cases remain under-diagnosed, which indicates that it is necessary to conduct better-developed studies to investigate the effect of depression screening on CLD management in this population. In previous studies, inconsistent findings regarding the prevalence and depression treatment outcomes in patients with CLD seem partly attributable to some limitations, including inconsistencies in the definition and measurement of depression, non-probability sampling and the absence of group matching. Further research is needed to identify depression in large population-based prospective cohorts of patients with CLD, comparing them with healthy controls, with patients with other long-term disorders, across the life course.

Relative to many studies of the prevalence of depression in patients with CLD, mechanistic studies of depression have been sparse. Generally, the main reasons include the following aspects: (i) the disease itself: the long-term pain caused by disease and treatment, sexual dysfunction and feeling of guilt, worry about disease progression, and fear of infecting family and friends, etc. (ii) social and economic pressure, including fundamental conditions for studying and working, social discrimination and high cost of medical treatment. (iii) Drug therapy: IFN has been widely used to treat viral hepatitis, in the meantime, depression is much more prevalent in CLD patients with IFN treatment than that in patients without it. However, these are the common characteristics of all chronic diseases, which cannot explain why CLD patients have a high prevalence of depression. For example, the consensus suggests that worsened depression symptoms are noted in patients with CH-C, but whether these symptoms consistently correlate with poor liver function is still not fully elucidated, and knowledge of mechanisms for depression in patients with CH-C is limited. Additionally, it is still unknown whether depression could alter the liver tissue structurally and functionally. Further research needs to identify some biomarkers in both biological processes, to elucidate the potential biological correlations between depression and CLD. Large, well characterized cohorts are needed to test whether shared origins of depression and CLD exist at a genome-wide association significance level.

## Author Contributions

XH designed the topic and wrote the manuscript. XL helped to searched references. YY helped to revise the manuscript. All authors approved the final manuscript.

## Conflict of Interest Statement

The authors declare that the research was conducted in the absence of any commercial or financial relationships that could be construed as a potential conflict of interest.

## Abbreviations

5-HTR1A: serotonin 1A receptor
5-HTTLPR: serotonin transporter gene-linked polymorphic region
ACTH: adrenocorticotropic hormone
ALD: alcoholic liver disease
BDI-sf: Beck Depression Inventory Short Form
BDNF: brain-derived neurotrophic factor
BMI: body mass index
CH-B: chronic hepatitis B
CH-C: chronic hepatitis C
CLD: chronic liver disease
CNS: central nervous system
DSM: Diagnostic and Statistical Manual of Mental Disorders
GAF: Global Assessment of Functioning
GGT: γ-glutamyl transpeptidase
GHQ: General Health Questionnaire
HBsAg: hepatitis B surface antigen
HBV: hepatitis B virus
HCC: hepatocellular carcinoma
HCV: hepatitis C virus
HPA: hypothalamic–pituitary–adrenal
HRQOL: health-related quality of life
HSC: hepatic stellate cell
hs-CRP: high-sensitivity C-reactive protein
IDO: indoleamine-2,3-dioxygenase
IFN: Interferon
IL: interleukin
KCs: kupffer cells
LT: liver transplantation
MDD: major depressive disorder
MDE: major depressive episode
NAFLD: non-alcoholic fatty liver disease
NASH: non-alcoholic steatohepatitis
NLRs: NOD-like receptors
NLRP3: NLRs family pyrin domain containing 3
NOD: nucleotide-binding oligomerization domain
PET: positron emission tomography
RBV: ribavirin
TRP: tryptophan
